# Population analysis of the Korean native duck using whole-genome sequencing data

**DOI:** 10.1186/s12864-020-06933-z

**Published:** 2020-08-12

**Authors:** Daehwan Lee, Jongin Lee, Kang-Neung Heo, Kisang Kwon, Youngbeen Moon, Dajeong Lim, Kyung-Tai Lee, Jaebum Kim

**Affiliations:** 1 Department of Biomedical Science and Engineering, Konkuk University, Seoul, 05029 Republic of Korea; 2 National Institute of Animal Science, Wanju, 55365 Republic of Korea

**Keywords:** Korean duck, Population analysis, Single nucleotide polymorphism

## Abstract

**Background:**

Advances in next-generation sequencing technologies have provided an opportunity to perform population-level comparative genomic analysis to discover unique genomic characteristics of domesticated animals. Duck is one of the most popular domesticated waterfowls, which is economically important as a source of meat, eggs, and feathers. The objective of this study is to perform population and functional analyses of Korean native duck, which has a distinct meat flavor and texture phenotype, using whole-genome sequencing data. To study the distinct genomic features of Korean native duck, we conducted population-level genomic analysis of 20 Korean native ducks together with 15 other duck breeds.

**Results:**

A total of 15.56 million single nucleotide polymorphisms were detected in Korean native duck. Based on the unique existence of non-synonymous single nucleotide polymorphisms in Korean native duck, a total of 103 genes related to the unique genomic characteristics of Korean native duck were identified in comparison with 15 other duck breeds, and their functions were investigated. The nucleotide diversity and population structures among the used duck breeds were then compared, and their phylogenetic relationship was analyzed. Finally, highly differentiated genomic regions among Korean native duck and other duck breeds were identified, and functions of genes in those regions were examined.

**Conclusions:**

This is the first study to compare the population of Korean native duck with those of other duck breeds by using whole-genome sequencing data. Our findings can be used to expand our knowledge of genomic characteristics of Korean native duck, and broaden our understanding of duck breeds.

## Background

In recent years, next-generation sequencing (NGS) technologies have dramatically improved in terms of cost, speed, and productivity [1]. This trend has provided us novel opportunities for large-scale population-level genome analysis. As a result, many population-level genome projects, such as the 1000 bull genomes project [2], Bird 10 K project [3], and 100,000 genomes project [4], have been launched. Recently, many population-level studies for various species have also been conducted to identify unique genomic features of a specific population of interest. For example, analysis using sequencing data of a total of 89 individuals in polar bear and brown bear populations was conducted to identify the divergence point of the two bear breeds [5]. Different genomic characteristics related to extreme environment adaptation have been studied for 77 individual sheep using whole-genome sequencing data [6]. Sequencing data of 57 platypuses living across eastern mainland Australia and Tasmania were used to uncover their dispersal and demographic history [7]. Also, various comparative analyses using whole-genome sequencing data have been performed to compare wild and domestic animal populations such as dog [8], pig [9], and chicken [10].

The duck is one of the most common domesticated waterfowls and is economically important as a source of meat, eggs, and feathers [11]. As a result, various genetic studies have been conducted to discover economically valuable genetic characteristics of duck breeds. For example, three duck breeds, which were artificially selected in China, were analyzed to identify the genetic features related to artificial selection based on whole-genome sequencing data [12]. Positively selected genes and differentially expressed genes involved with muscle growth and lipid deposition were identified by comparing native Pekin duck and Cherry Valley Pekin duck using whole-genome and transcriptome sequencing data [13].

**Fig. 1:**
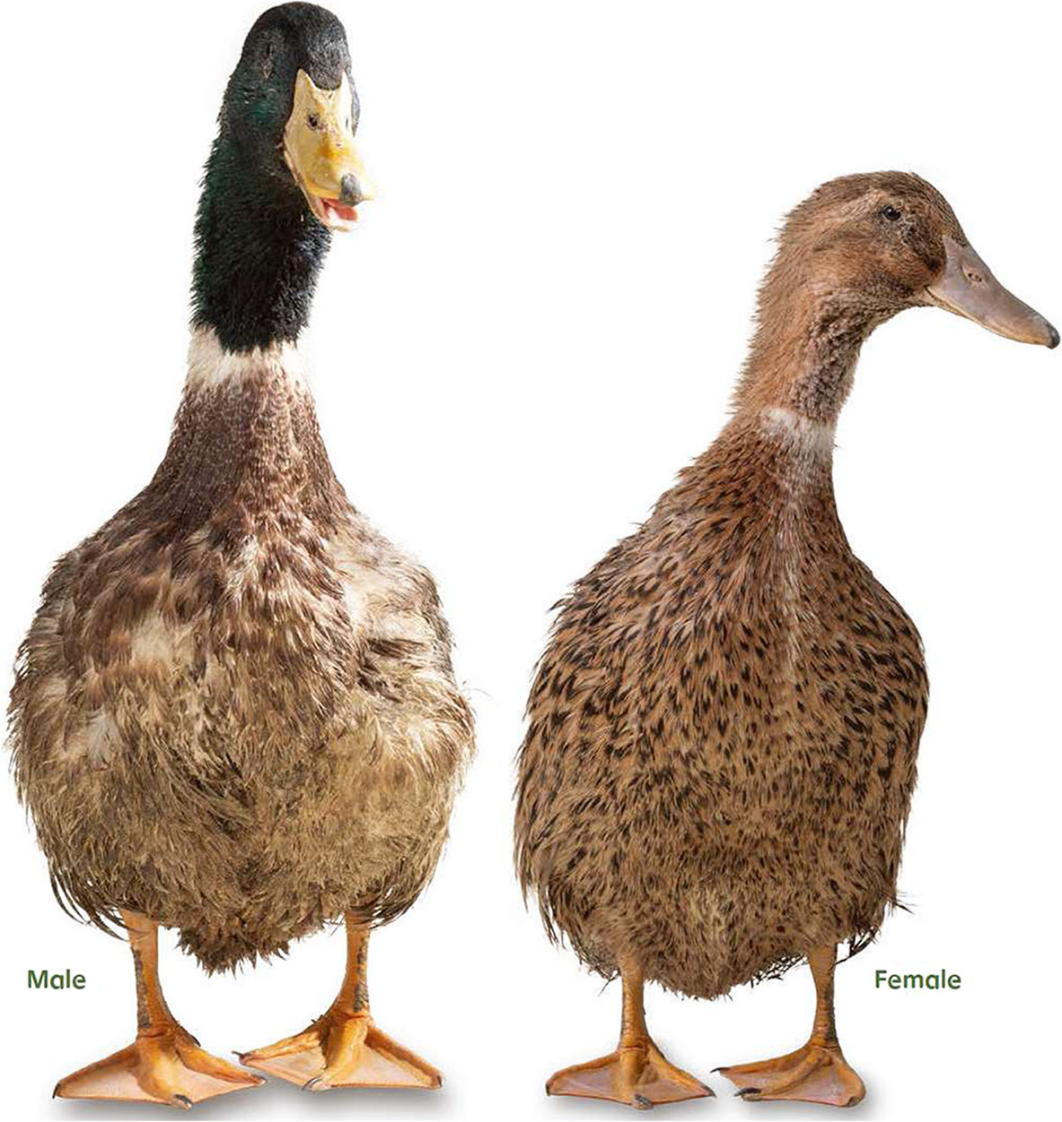
Appearance of male and female Korean native duck. Pictures were obtained from the Poultry Research Institute, and the Animal Genetic Resources Research Center in Republic of Korea

Korean native duck (KD), called Woorimatori, is a domesticated duck that originated from the hybridized ducks between mallard duck and indigenous Pekin duck, and has been continuously improved since 1997 at National Institute of Animal Science, Republic of Korea by selecting individuals with excellent appearance, weight, and productivity [14–16]. They resemble the appearance of a mallard duck with glossy dark brown feathers, and a dark green head in males (Fig. 1 and more characteristics in Additional file 1: Table S1). In addition, they are general purpose type duck that has excellent economic efficiency and productivity. They have a high crude protein, water retention capacity, and unique meat flavor and texture with high polyunsaturated fatty acids in breast meat and high essential fatty acids, arachidonic acid [17]. Although some recent studies have investigated the unique characteristics of the Korean native duck [17–20], whole genome-level studies for the Korean native duck still lag behind other domestic animals and duck breeds.

To address this, we apply a population-level genome analysis based on whole-genome sequencing data from populations of various duck breeds including KD. Specifically, we sequenced the whole genomes of 20 KDs, collected whole-genome sequencing data of 14 phenotypically diverse duck breeds (Additional file 1: Table S1), and discovered single nucleotide polymorphisms (SNPs) for 15 duck breed populations including KD. We discovered candidate genes related to the unique characteristics of KD based on the existence of non-synonymous SNPs (nsSNPs) compared to nsSNPs of other breeds. Additionally, we examined the population structure of 15 duck breeds using various methodologies such as principal component analysis (PCA), admixture, and phylogeny estimation. We also identified genomic regions under high differentiation among duck breeds and performed functional analysis of genes in those regions. Our findings provide extensive knowledge of KDs and proved an example of comprehensive analysis using whole-genome sequencing data for native animals.

## Results

### Genome resequencing, SNP calling and annotation

We performed whole-genome resequencing of 20 Korean native ducks (KDs) at a mean coverage of 25.7x (see Methods; Additional file 2: Table S2), and detected single nucleotide polymorphism (SNP) for 123 individuals of 15 duck breeds (see Methods). We also annotated SNPs and summarized the results for 15 duck breeds. A total of 15,557,752, 14,629,071, and 28,920,088 SNPs were discovered from KD, Pekin duck (PK), and mallard duck (MD), respectively (Table 1). We also identified the number of SNPs in indigenous duck breeds (Longsheng (LS), Jiding (JD), Loancheng white (LC), Mawang (MW), Puitan black (PT), Shan (SM), Sansui (SS), Shaoxing (SX), Taiwan (TW), Youxian (YX), Ji’an red (JA), and Gaoyou (GY)), which ranged from 8,787,171 to 10,667,745. We calculated transition to transversion (Ti/Tv) ratios to assess the overall SNP quality. The Ti/Tv ratio for KD, PK, and MD were 2.53, 2.51 and 2.51, respectively, and for the indigenous duck breeds have shown the Ti/Tv ratio ranging from 2.53 to 2.56. We annotated all SNPs for 15 duck breeds with 19 functional categories, including synonymous, non-synonymous, intron, untranslated regions, and intergenic (Additional file 3: Table S3).

**Table 1:** SNP statistics of 15 duck breeds

Duck breed	No. of SNPs	Ti/Tv ratio^a^
Korean native duck (KD)	15,557,752	2.53 (0.0045)
Mallard duck (MD)	28,920,088	2.51 (0.0131)
Pekin duck (PK)	14,629,071	2.51 (0.0231)
Longsheng Cui-duck (LS)	9,616,438	2.55 (0.0180)
Jinding duck (JD)	9,012,541	2.53 (0.0110)
Liancheng white duck (LC)	8,787,171	2.56 (0.0127)
Mawang duck (MW)	10,702,303	2.56 (0.0164)
Putian black duck (PT)	10,704,884	2.56 (0.0148)
Shan sheldrake (SM)	9,917,514	2.55 (0.0183)
Sansui duck (SS)	10,667,745	2.56 (0.0192)
Shaoxing duck (SX)	10,446,665	2.55 (0.0171)
Taiwan sheldrake (TW)	9,123,754	2.55 (0.0124)
Youxian sheldrake (YX)	10,644,149	2.56 (0.0214)
Ji’an red duck (JA)	10,061,461	2.55 (0.0181)
Gaoyou duck (GY)	9,980,748	2.55 (0.0181)

^a^Ti/Tv ratio is the ratio of the number of transitions to the number of transversions and standard deviations are in parentheses

### Investigation of unique genomic characteristics of Korean native duck

**Fig. 2:**
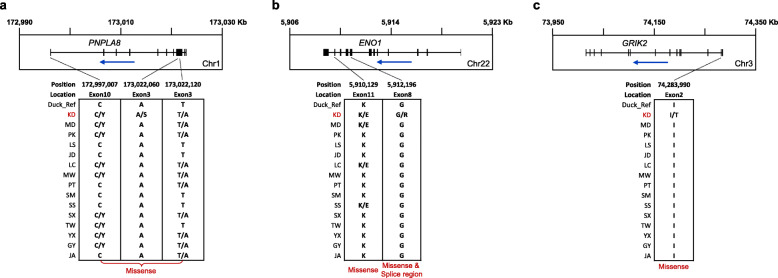
Examples of KD genes **(a** and **b)** and KD-specific gene **(c)**. Top panel shows gene structure with the direction of transcription (blue arrow). Bottom panel indicates positions of non-synonymous SNPs and comparison of amino acids among different duck breeds. Note that the positions of bottom panel in A and B represent examples among all nsSNP positions. Below the bottom panel indicates sequence ontology predicted by SnpEff. Two different amino acids corresponding to two nucleotide variants are shown together with a slash delimiter. Duck_Ref: IASCAAS_PekingDuck_PBH1.5, Korean native duck: KD, Pekin duck: PK, mallard duck: MD, Gaoyou duck: GY, Longsheng Cui-duck: LS, Shaoxing duck: SX, Ji’an red duck: JA, Sansui duck: SS, Putian black duck: PT, Mawang duck: MW, Youxian sheldrake: YX, Shan sheldrake: SM, Jinding duck: JD, Taiwan sheldrake: TW and Liancheng white duck: LC

To investigate the unique genomic characteristics of the Korean native duck (KD), we found 3062 KD genes containing unique non-synonymous SNPs (nsSNPs) not observed at the same position in other duck breeds (see Methods; Additional file 4: Table S4). We performed Gene Ontology (GO) enrichment analysis to find potential functions for these genes (see Methods). Among the various biological processes, multicellular organismal process (GO:0032501), developmental process (GO:0032502), systems development (GO:0048731), anatomical structure development (GO:0048856), cellular component organization or biogenesis (GO:0071840), multicellular organism development (GO:0007275), and cellular component organization (GO:0016043), were found to be highly enriched (Additional file 5: Table S5). Enriched GO terms in the molecular function and cellular component category are also shown in Additional file 5: Table S5. In addition, we examined how the amino acid composition of these genes differs from other duck breeds. Among the 3062 genes, two genes (*PNPLA8* and *ENO1*) are shown as examples in Fig. 2. In the case of *PNPLA8* (Fig. 2a), only KD had serine as an alternative allele with alanine as a reference allele, caused by a G > T nsSNP at the position of 173,022,060. At other positions of *PNPLA8* (e.g. 172,992,007 and 173,022,120), several breeds including KD had different amino acids as alternative allele caused by missense variant. Similarly, the *ENO1* gene had a locus which exhibited a G > C nsSNP only in KD. This G > C nsSNP at position 5,912,196 in the eighth exon leads to arginine as an alternative allele with glycine as a reference allele in KD (Fig. 2b). The *ENO1* gene also had other positions where amino acid changes occur due to missense variant in various duck breeds.

We further filtered the above KD genes and obtained KD-specific genes which have only KD unique nsSNPs, not with nsSNPs of the other duck breeds (see Methods). A total of 103 KD-specific genes were found (Additional file 6: Table S6). We also conducted Gene Ontology enrichment analysis for these genes, but there are no significantly enriched functions. Among them, however, *GRIK2* known to be related with domestication [21] was included in the gene list. Figure 2c shows an example of the change of amino acid composition in the *GRIK2* gene. The T > C nsSNP in this gene led to threonine as an alternative allele with isoleucine as a reference allele, which has only occurred in KD.

### Nucleotide diversity, population structure and phylogenetic relationship analysis

**Fig. 3:**
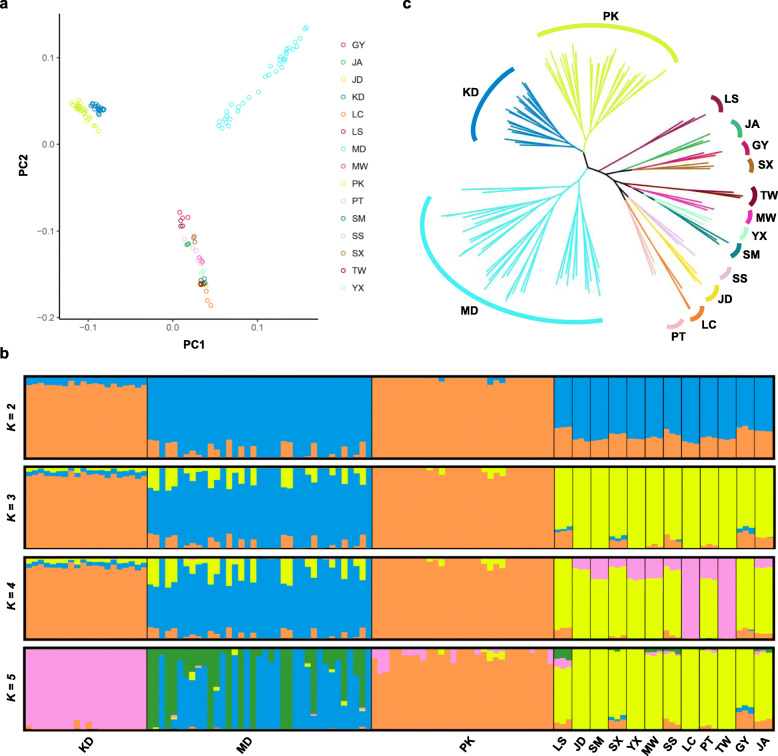
Population structure analysis of 15 duck populations (Pekin duck: PK, mallard duck: MD, Gaoyou duck: GY, Longsheng Cui-duck: LS, Shaoxing duck: SX, Ji’an red duck: JA, Sansui duck: SS, Putian black duck: PT, Mawang duck: MW, Youxian sheldrake: YX, Shan sheldrake: SM, Jinding duck: JD, Taiwan sheldrake: TW and Liancheng white duck: LC). **a** The principal component analysis plot of 15 duck populations with the first two components. **b** Population genomic structures obtained by the number of clusters *K* (from 2 to 5). Each individual is represented with a vertical line. The length of each colored segment represents a relative membership to different clusters. **c** Maximum-likelihood phylogenetic tree of 15 duck populations. Color of each branch corresponds to the color in the PCA plot for each duck population

After filtering out SNPs using various criteria such as minor allele frequency, genotype rate and the Hardy-Weinberg equilibrium (see Methods), we obtained a total of 8,769,869 SNPs from 123 individuals of 15 duck breeds. We first calculated the mean nucleotide diversity (π) [22] for each of 15 duck breeds using the filtered SNPs (Table 2; Methods). MD showed the highest π value (0.1698), which is clearly larger than the values of other duck breeds (from 0.1028 to 0.1384). The lowest π value was observed in LC (0.1028), and the π value of KD was 0.1338 which is higher than PK (0.1221). We next used two approaches to identify the population structure of 15 duck breeds. First, we conducted principal component analysis (PCA) to identify genomic relationships among 15 duck breeds. By the first two principal components, 15 duck breeds were divided into three major clusters (Fig. 3a). KD and PK breeds were very tightly clustered together, whereas the MD breed was loosely stretched. The remaining cluster included all indigenous breeds (LS, JD, SM, SX, YX, MW, SS, LC, PT, TW, GY, and JA). More detailed relationships between the other principal components are provided in Additional file 7: Fig. S1. Second, we analyzed the population structure of 15 duck breeds using ADMIXTURE to estimate admixture proportion and individual ancestry based on the called genotypes (see Methods; Fig. 3b). At K = 2, similar to the results of PCA, KD and PK were distinguished from the rest of the breeds. Additionally, from the results at K = 3, we identified a division between MD and all indigenous breeds. When K = 5, we found that KD and PK were separated and observed a subdivision in MD. At K = 5, we observed genomic relationships among the 15 duck breeds consistent with the results of PCA. We then constructed a maximum likelihood tree using a subset of 12,566 high-quality SNPs to identify the phylogenetic relationships among 15 duck breeds (Fig. 3c). We confirmed that most of the individuals in the same breeds were grouped into one cluster, and these results were also consistent with the PCA result in terms of the first two principal components (Fig. 3a).

**Table 2:** Nucleotide diversity (π) of 15 duck breeds

Duck breed	Mean nucleotide diversity (Stdev)
Korean native duck (KD)	0.1338 (0.0325)
Mallard duck (MD)	0.1698 (0.0267)
Pekin duck (PK)	0.1221 (0.0306)
Longsheng Cui-duck (LS)	0.1210 (0.0444)
Jinding duck (JD)	0.1111 (0.0426)
Liancheng white duck (LC)	0.1028 (0.0404)
Mawang duck (MW)	0.1384 (0.0481)
Putian black duck (PT)	0.1379 (0.0479)
Shan sheldrake (SM)	0.1262 (0.0463)
Sansui duck (SS)	0.1383 (0.0480)
Shaoxing duck (SX)	0.1341 (0.0471)
Taiwan sheldrake (TW)	0.1103 (0.0424)
Youxian sheldrake (YX)	0.1364 (0.0476)
Ji’an red duck (JA)	0.1288 (0.0465)
Gaoyou duck (GY)	0.1276 (0.0460)

### Population differentiation analysis

**Fig. 4:**
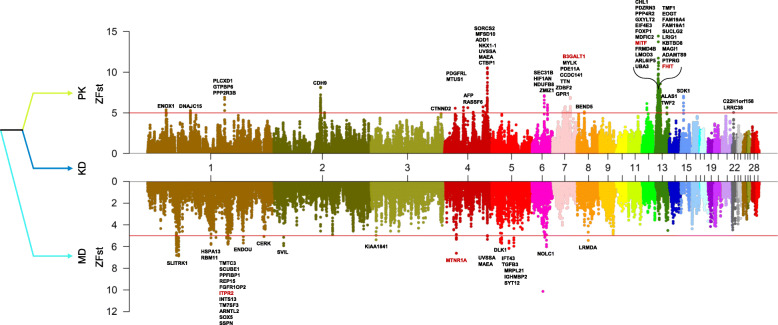
Manhattan plot of Z-transformed Fst (ZFst) between Korean native duck and Pekin (top panel) and between Korean native duck and mallard duck (bottom panel) in sliding window 40 Kb with 10 Kb steps across the autosomal chromosomes. Red line denotes a threshold of ZFst at 5. Genes located in differentiated genomic regions are indicated by their gene symbols

To identify the differentiated genomic regions among duck populations, we calculated the Z-transformed Fst (ZFst) values based on SNPs in 40 Kb sliding genomic regions with 10 Kb steps (see Methods). We investigated the population differentiation among the KD, PK, and MD populations based on the estimated tree topology (Fig. 4 and Additional file 8: Fig. S2). In total, we identified 309 and 107 highly differentiated genomic regions (ZFst > 5) with 101 and 54 genes across autosomal chromosomes for the KD versus PK population and the KD versus MD population, respectively. In the case of the KD versus PK population, some highly differentiated regions included the *MITF* gene related to melanocyte differentiation (GO:0030318) and pigmentation (GO:0043473), and the *B3GALT1* gene associated with lipid glycosylation (GO:0030259) (Fig. 4). In the case of the KD versus MD population, the *MTNR1A* and *ITPR2* genes were observed in highly differentiated regions (Fig. 4). These genes were related to melatonin receptor activity (GO:0008502), and calcium-release channel activity (GO:0015278), and inositol 1,4,5-trisphosphate-sensitive calcium-release channel activity (GO:0005220), respectively. Additionally, we identified 99 highly differentiated genomic regions with 53 genes for the KD versus other 14 duck population (Additional file 9: Fig. S3). In this case, genes related with hydrolase activity (GO:0016787) and growth factor activity (GO:0008083), such as *ABHD17A* and *TGFB3*, were observed in several differentiated genomic regions.

## Discussion

In this study, we performed whole-genome resequencing of 20 individual Korean native ducks (KD) using high-throughput next-generation sequencing technologies, and conducted a comparative analysis with 14 duck breeds based on single nucleotide polymorphism (SNP) data. The 14 duck breeds were selected because they are well categorized according to their phenotypes, and their relationship among Pekin duck (PK), mallard (MD), and indigenous duck breeds is well studied [23].

Similar to previous studies [24–26], we called SNPs of each duck breed by mapping sequencing data to a duck reference genome, and applying various filtering steps, such as duplicate read handling, local realignment, and removal of low-quality calls, to ensure the high-quality of SNPs. We detected and annotated a total of 51,154,530 high-quality SNPs from the 15 duck breeds, and identified two types of gene sets based on the existence of non-synonymous SNPs (nsSNPs) in order to find unique genetic characteristics of the KD breed compared to other duck breeds. One is the KD genes which contain loci where only KD has nsSNPs (but can have loci with nsSNPs only found in other breeds), and another is the KD-specific genes which have loci with nsSNPs only found in KD. Among the 3062 KD genes, the *PNPLA8* gene is involved in energy mobilization and lipid storage in adipocyte tissue [27], and the *ENO1* gene plays a role in the glycolysis pathway as an enzyme which is related to fatty acid synthesis [28]. Although no significantly enriched function has been identified in the 103 KD-specific genes, these genes may underlie a difference between KD and other breeds. For example, the *GRIK2* gene, which encodes a subunit of a glutamate receptor, (i) has a crucial role in synaptic plasticity, (ii) is involved in learning and memory, and (iii) plays an important role during rabbit domestication [21].

The mean nucleotide diversity (π) of duck breeds was between 0.1028 to 0.1384, and MD showed exceptionally higher nucleotide diversity than domesticated breeds. KD has a higher nucleotide diversity (0.1338) than PK (0.1221), which may be because the breeding history of KD (since late 1990s) is shorter than PK (since the Ming Dynasty) [14, 23]. We also performed population analyses of 15 duck breeds including KD, and found similar patterns for duck breeds used in the previous study [23]. In addition, our results show that the KD breed has a close genomic relationship and shared admixture history with the PK breed. This can be explained by the domesticaton history of KD, which was formed with hybridized ducks of indigenous PK and wild mallard duck in the late 1990s and has been improved to current KD (Woorimatori) population [14, 16].

We conducted differentiation analysis to discover what makes the difference among KD, PK, and MD breeds, and identified several candidate regions covering highly differentiated SNPs with respect to KD versus PK and KD versus MD. In the highly differentiated regions between KD and PK, we found several phenotype-related genes such as *B3GALT1*, *FHIT*, and *MITF*. The *B3GALT1* gene is related to lipid glycosylation (GO:0030259) which affects lipid accumulation [29]. The *FHIT* gene is associated with body mass index [30]. The *MITF* gene plays an important role in the melanogenesis pathway [31, 32] and is involved in functions such as melanocyte differentiation (GO:0030318) and pigmentation (GO:0043473). This gene may explain why KD has glossy dark brown feathers while PK has white plumage [14, 23]. Also, we found two interesting genes, *MTNR1A* and *ITPR2*, in highly differentiated regions between KD and MD. Sequential genetic variation in the *MTNR1A* gene is associated with the reproductive behavior of a local Greek sheep breed and goat [33, 34], and also some SNPs in this gene may affect duck reproduction [35]. The *ITPR2* gene plays a crucial role in the regulation of intracellular calcium transportation and the process of eggshell calcification related to eggshell quality [36]. We suggest that these genes in the highly differentiated regions could be candidates for improving reproductivity, meat quality, and egg quality. Additionally, we investigated differentiated genomic regions among KD and other 14 duck breeds, and found 53 genes including *ABHD17A*, *SSH2* and *TGFB3* associated with palmitoyl-(protein) hydrolase activity (GO:00008474), hydrolase activity (GO:0016787) and growth factor activity (GO:0008083). Genes related with these functions may have created difference between KD and other 14 duck breeds. Zhou et al. compared populations of MD, PK, and indigenous-breed ducks, and found a regulatory mutation in a long-distance upstream region of the *IGF2BP1* gene [23]. The long-distance mutation may have a potential to induce continuous expression of the *IGF2BP1* gene, which is related to large body size in PK. The long-distance regulatory region and the *IGF2BP1* gene were not included in the differentiated genomic regions obtained from the comparison between KD versus PK and KD versus MD in our study.

## Conclusions

In summary, our study represents the first population-level analysis of 15 duck breeds including Korean native duck (KD) based on whole-genome sequencing data. Our results include candidate genes associated with unique characteristics of KD, and the genetic relationship among the 15 duck breeds. As a result, our research provides a comprehensive overview of the population structure and genetic diversity of 15 duck breeds, and will help further investigate the genetic information underlying commercially valuable traits in the KD breed.

## Methods

### Sequencing and library preparation

We generated whole-genome resequencing data from a population of Korean native duck (KD; *N* = 20). The Korean duck samples were collected from Myeongbawinongsan (Yongin, Korea) in compliance with relevant guidelines, using protocols approved by the Committee on the Ethics of Animal Experiments of the National Institute of Animal Science (Permit Number: NIAS2015–775). Each sequenced sample was prepared according to the Illumina protocols (TruSeq DNA Sample Prep Kit v2 Support (FC121–2001)). Briefly, one microgram of genomic DNA was fragmented by Covaris, the fragmented DNA is repaired, and an ‘A’ is ligated to the 3′ end. Illumina adapters are then ligated to the fragments, and the sample is size selected aiming for 400 ~ 500 base pair products. The size selected product is PCR amplified, and the final product validated using the Agilent Bioanalyzer. After that, selected DNA was sequenced using the HiSeq2000 platform (Illumina, San Diego, USA) by Macrogen (Seoul, Republic of Korea).

### Read alignment and variant calling

To generate single nucleotide polymorphism (SNP) data, we collected public sequencing data of various breeds of duck (Pekin; PK (*N* = 30), Mallard; MD (*N* = 37), and twelve Chinese indigenous breeds; Gaoyou (GY), Longsheng (LS), Shaoxing (SX), Ji’an red (JA), Sansui (SS), Putian black (PT), Mawang (MW), Youxian (YX), Shan (SM), Jinding (JD), Taiwan (TW) and Liancheng white (LC) (N = 3 for these breeds)) from the NCBI SRA database (https://www.ncbi.nlm.nih.gov/sra; Additional file 2: Table S2 for accession numbers of the data). A total 103 public sequencing data of various duck breeds and 20 resequencing data of KD were aligned to the chromosome-level duck reference genome (assembly version IASCAAS_PekingDuck_PBH1.5; accession number GCF_003850225.1) downloaded from the NCBI RefSeq database [37] using BWA-MEM (v0.7.17) with default parameters [38]. After aligning, SAMtools (version 1.3.1) was used for converting SAM to BAM format, sorting, and indexing process [39]. Filtering of duplicate reads which mapped to the same position on the reference genome, and generation of quality matrices for mapping were processed using the MarkDuplicates program in the Picard tool (v2.17.11; http://broadinstitute.github.io/picard). Local realignment was performed using the Genome Analysis ToolKit (GATK v3.8.1) tool [40]. Because publically available duck SNPs did not yet exist, the first SNP calling procedure was performed using HaplotypeCaller without the recalibration step. The output was filtered as follows: “QD < 2.0, MQ < 40.0, FS > 60.0, MQRankSum < -12.5, ReadPosRankSum < -8.0”. Then, the recalibration step was performed with filtered SNPs as the database of known SNPs, and the second SNP calling procedure proceeded with recalibrated data using HaplotypeCaller. Finally, raw calling data was filtered using the same criteria as in the previous filtering step.

### SNP annotation, KD-specific gene identification, and functional analysis

We built a database with the NCBI RefSeq gene annotation data (duck annotation release 103) of the reference duck assembly (IASCAAS_PekingDuck_PBH1.5) [37], and performed variant annotation for the final SNPs of 15 duck breeds using SnpEff v4.3 [41]*.* We also calculated the transition-to-transversion ratio (Ti/Tv) to evaluate the quality of the SNPs. Using the annotated SNP information, KD genes with unique non-synonymous SNPs (nsSNPs), which were not observed at the same position in other duck breeds, were identified. Note that these genes can have unique nsSNPs of other duck breeds or common nsSNPs among other duck breeds. Therefore, we further reduced those KD genes to KD-specific genes which have only KD unique nsSNPs, not with nsSNPs of other duck breeds. Functional analysis of the above KD genes and KD-specific genes was performed by g:Profiler with default parameters [42].

### Nucleotide diversity, population structure and selective sweep analysis

SNP data was filtered with PLINK (v1.90) using the following criteria: “--geno 0.01 –maf 0.05 --hwe 0.000001” [43]. For each duck breed, the nucleotide diversity (π) was calculated for each of 40 Kb sliding genomic windows (with 10 Kb steps) using the filtered SNPs by the populations program in Stacks (v2.53) with default parameters [44]. In this calculation, only autosomal chromosomes were used, and mean nucleotide diversity from all genomic windows were reported. Principal component analysis (PCA) was performed using GCTA (v1.24.4) [45]. First, a genetic relationship matrix was calculated with the “--make-grm” option, and then four principal components were estimated with the “--pca 4” option. The ggplot2 R package was used to visualize the PCA plot [46]. The ancestry of each individual was estimated by ADMIXTURE (v1.3.0) [47] with 200 bootstrap replicates and the number of ancestral clusters K ranging from 2 to 6. The estimated ancestry for each cluster was visualized by CLUMPAK [48]. A phylogenetic tree was constructed based on the SNPs filtered by PLINK (v1.90) with “--indep-pairwise 50 5 0.2” option to reduce SNP redundancy caused by linkage disequilibrium using SNPhylo [49]. A total of 12,566 high-quality SNPs were used to build a maximum likelihood phylogenetic tree using SNPhylo with default parameters, and 1000 bootstrap replicates. To investigate differentiated regions among the various duck populations, the mean Fst value was calculated using VCFtools (v.0.1.13) [50] for 40 Kb sliding genomic windows with 10 Kb steps in autosomal chromosomes, and it was Z-transformed as follows: ZFst = (Fst – *μ* Fst)/ *σ* Fst, where Fst is the Fst in a window, *μ* Fst is an average Fst over all windows, and *σ* Fst is a standard deviation of Fst values of all windows [51]. Genes in the genomic regions with high Z-transformed Fst value (> 5) were used to identify their functions in terms of gene ontology. The results of population differentiation were visualized in the form of a Manhattan plot by the qqman R package [52]. Functional analysis was performed by g:Profiler with default parameters [42].

## Supplementary information

**Additional file 1: Table S1.** Phenotypic information of 15 duck breeds.

**Additional file 2: Table S2.** Read mapping, coverage statistics and SRA accession number of 123 duck individuals.

**Additional file 3: Table S3.** Functional categories of annotated SNPs of 15 duck breeds.

**Additional file 4: Table S4.** A list of 3062 genes containing locus where only KD had non-synonymous SNPs compared to other duck breeds.

**Additional file 5: Table S5.** The results of gene set enrichment analysis of 3062 genes containing locus where only KD had non-synonymous SNPs.

**Additional file 6: Table S6.** A list of 103 genes containing only non-synonymous SNPs of KD.

**Additional file 7: Figure S1.** The principal component analysis plot of 15 duck populations for all pairs of four components.

**Additional file 8: Figure S2.** Maximum likelihood phylogenetic tree of 15 duck breeds with Muscovy duck as an outgroup. Color of each branch corresponds to the color in the PCA plot (Fig. 3a) for each duck population.

**Additional file 9: Figure S3.** Manhattan plot of Z-transformed Fst (ZFst) between Korean native duck and other 14 duck breeds in sliding window 40 Kb with 10 Kb steps across the autosomal chromosomes. Red line denotes a threshold of ZFst at 5. Genes located in differentiated genomic regions are indicated by their gene symbols.

## Data Availability

All SNPs of 15 duck breeds discovered in this study have been submitted to the European Variation Archive database (https://www.ebi.ac.uk/eva/; project ID: PRJEB34846).

## Abbreviations

NGS: Next-generation sequencing
SNP: Single-nucleotide polymorphism
Ti/Tv: Transition to Transversion ratio
nsSNP: non-synonymous SNP
GO: Gene ontology
PCA: Principal component analysis
Fst: Population-differentiation statistic
ZFst: Z-transformed Fst
