# Supplementary material for: Population analysis of the Korean native duck using whole-genome sequencing data
Source: BMC Genomics. 2020 Aug 12;21:554. doi: 10.1186/s12864-020-06933-z (PMC7430827; doi:10.1186/s12864-020-06933-z)
Supplement: Supplementary file 7 — Additional file 7: Figure S1. The principal component analysis plot of 15 duck populations for all pairs of four components. [file 12864_2020_6933_MOESM7_ESM.pdf]

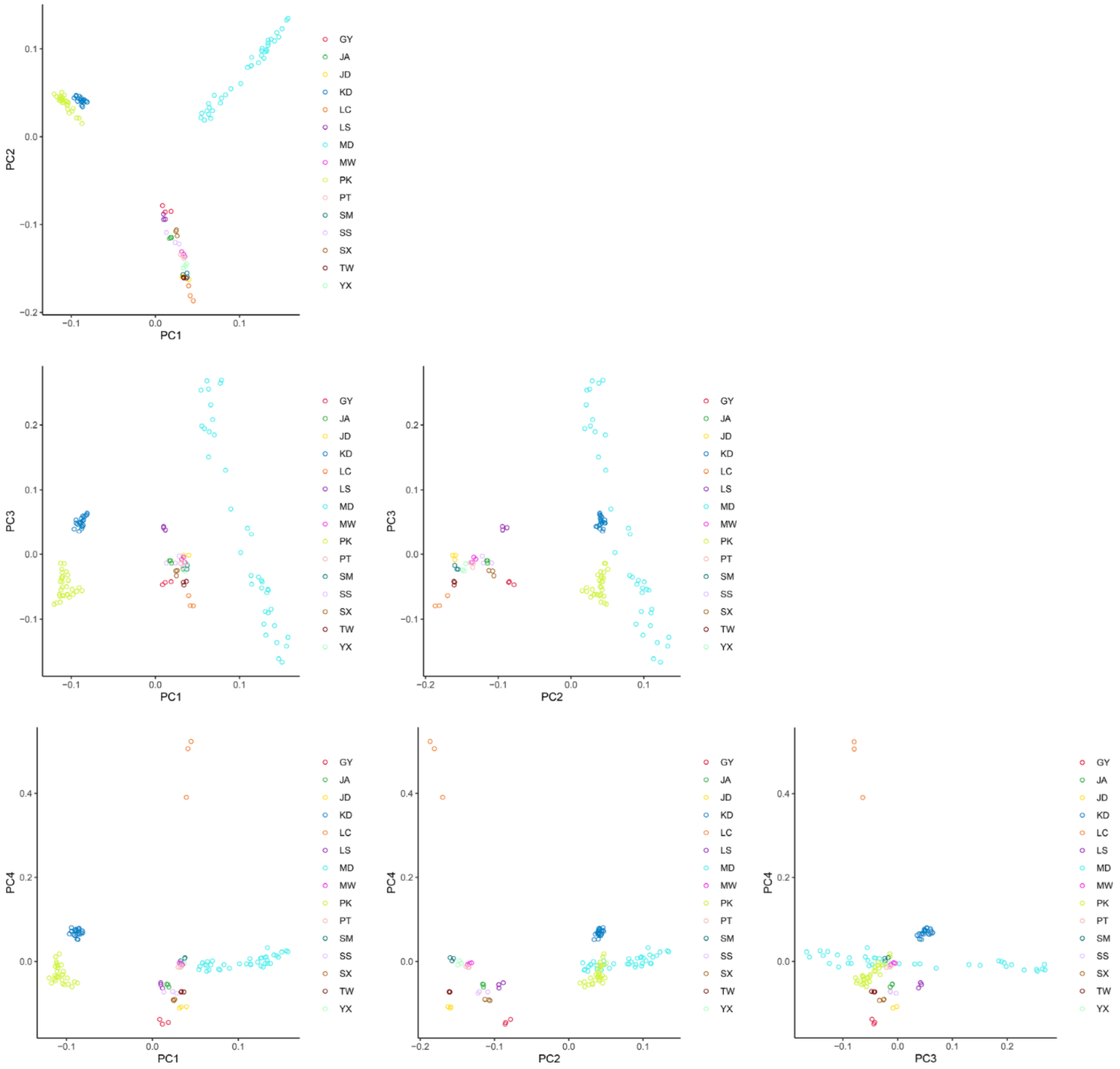

Additional file 7: Figure S1. The principal component analysis plot of 15 duck populations for all pairs of four components.
