# Supplementary material for: Population analysis of the Korean native duck using whole-genome sequencing data
Source: BMC Genomics. 2020 Aug 12;21:554. doi: 10.1186/s12864-020-06933-z (PMC7430827; doi:10.1186/s12864-020-06933-z)
Supplement: Supplementary file 8 — Additional file 8: Figure S2. Maximum likelihood phylogenetic tree of 15 duck breeds with Muscovy duck as an outgroup. Color of each branch corresponds to the color in the PCA plot (Fig. 3a) for each duck population. [file 12864_2020_6933_MOESM8_ESM.pdf]

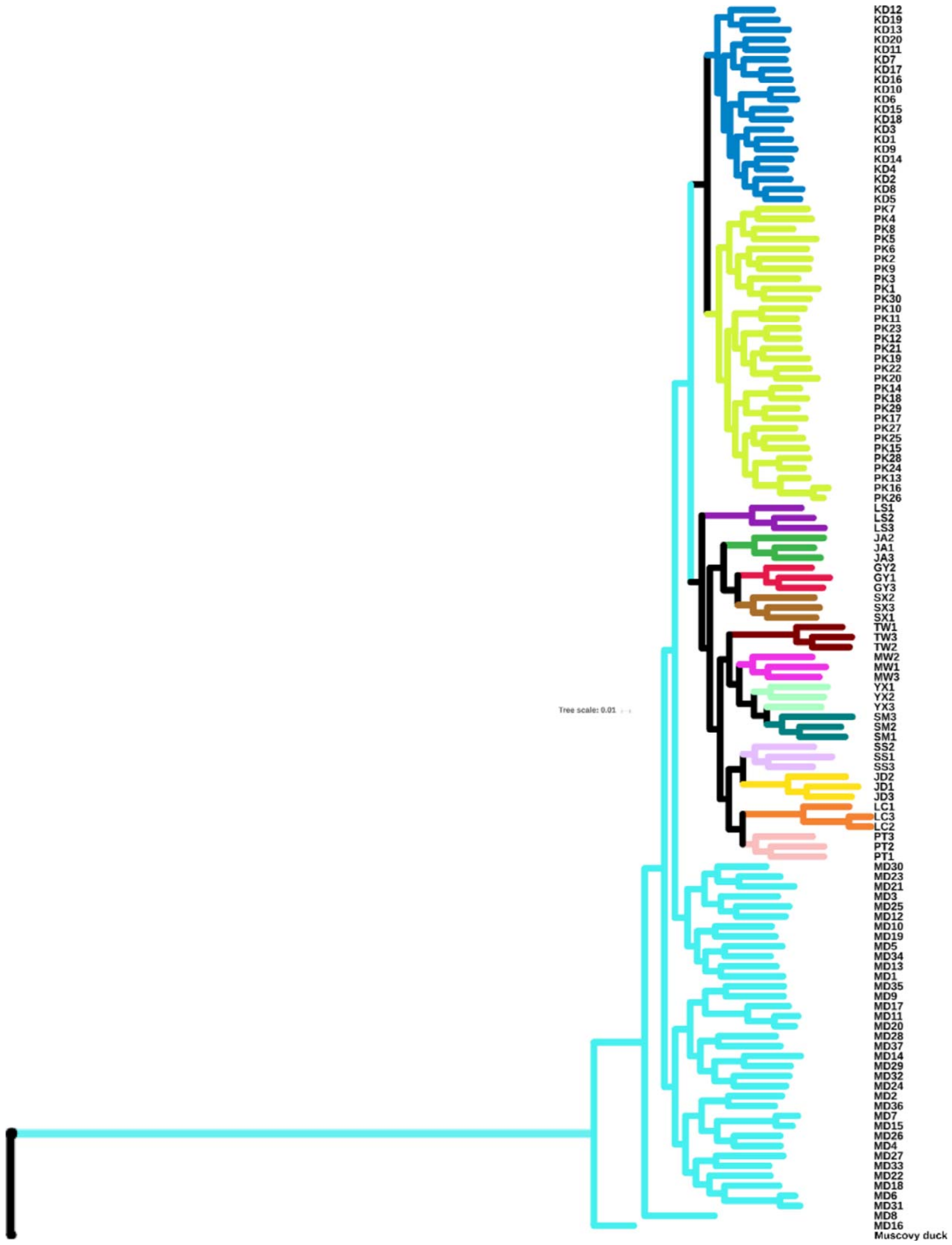

Additional file 8: Figure S2. Maximum likelihood phylogenetic tree of 15 duck breeds with Muscovy duck as an outgroup. Color of each branch corresponds to the color in the PCA plot (Fig. 2A) for each duck population.
