# Supplementary material for: Population analysis of the Korean native duck using whole-genome sequencing data
Source: BMC Genomics. 2020 Aug 12;21:554. doi: 10.1186/s12864-020-06933-z (PMC7430827; doi:10.1186/s12864-020-06933-z)
Supplement: Supplementary file 9 — Additional file 9: Figure S3. Manhattan plot of Z-transformed Fst (ZFst) between Korean native duck and other 14 duck breeds in sliding window 40 Kb with 10 Kb steps across the autosomal chromosomes. Red line denotes a threshold of ZFst at 5. Genes located in differentiated genomic regions are indicated by their gene symbols. [file 12864_2020_6933_MOESM9_ESM.pdf]

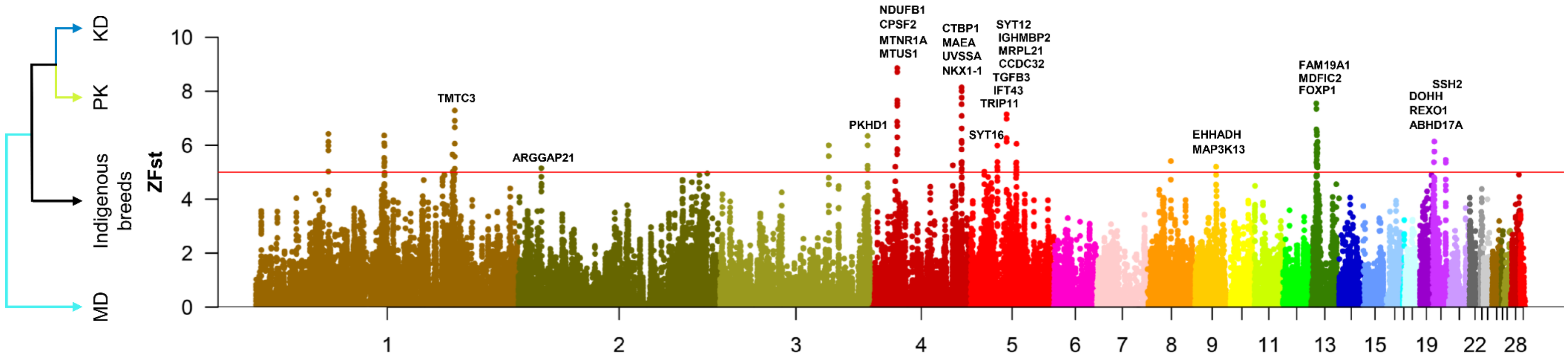

Additional file 9: Figure S3. Manhattan plot of Z-transformed Fst (ZFst) between Korean native duck and other 14 duck breeds in sliding window 40 Kb with 10 Kb steps across the autosomal chromosomes. Red line denotes a threshold of ZFst at 5. Genes located in differentiated genomic regions are indicated by their gene symbols.
